# Beyond broad and narrow: Intermediate level traits in the personality of bridge players

**DOI:** 10.1371/journal.pone.0305985

**Published:** 2024-08-22

**Authors:** Camille Sauvain, Véronique Ventos, Jérôme Sackur

**Affiliations:** 1 Laboratoire de Sciences Cognitives et Psycholinguistique (CNRS / ENS / EHESS), Paris, France; 2 NukkAI, Paris, France; 3 École Polytechnique, Palaiseau, France; COMSATS University Islamabad, PAKISTAN

## Abstract

Games offer a unique context for studying human behavior within the realm of social interactions, where a crucial aspect is the role of personality. The personality of individuals is often conceptualized as divided between general traits (broad traits) that are difficult to apply to specific situations and highly specific traits (narrow traits) that only offer a partial depiction of contextual aspects. In this study, we propose an intermediary level of traits revealed through self-ratings (as broad traits), but defined with respect to a particular game context (as narrow traits). We focus on the popular game of Bridge, which is complex and similar to real-life interactions involving incomplete information, adversarial and cooperative concerns, and communication between players. Using a multidimensional analysis of a new 66-item Bridge Inventory survey completed by 1,300 players, we identified five factors (Aggressiveness, Discipline, Creativity, Emotionality, and Experience) that were meaningfully correlated with broad traits of the Five Factor Model (FFM), supporting their validity. Based on these game-related traits, we identified three types of Bridge players: Conventional, Measured, and Subversive and demonstrated the limitations of FFM traits in capturing nuances of player types. The results of our study highlight a discrepancy between broad, context-independent personality traits and narrow, game-specific traits. We propose that this gap can be bridged through self-ratings, revealing a set of intermediate-level, context-dependent traits, which are expected to better encompass interindividual variability in the context of social interactions.

## Introduction

Games, especially economic games, have been used as a valuable tool, since the early 20th century, for studying human behavior in various contexts, encompassing realms such as warfare, economic exchanges, sports, political decisions, and beyond. Economic games provide a way to simulate complex social interactions while preserving experimental control [1, 2]. Moreover, these games directly elicit behaviors of agents facing “real” outcomes, increasing their motivation compared to other experimental methods [3]. Economic games showed a high variability in decision making among individuals (e.g., [1, 4, 5]). This variability challenges the predictions of theoretical models in game theory that assume agents are rational maximizers of their self-interests [6]. As a consequence, psychologists suspect that personality differences are implicated, as a major source of variability between individuals [7], in the process of decision making involved in economic games [8].

In the existing literature on the effect of personality in games, two distinct directions can be identified. On one hand, some psychologists have measured the impact of broad personality traits that are defined *a priori* and not derived from in-game measures, on behaviors in economic games. Indeed, the Five Factor Model [9, 10] and the HEXACO constructs [11, 12], which are the two main models of broad personality traits, have been developed using a-contextual self-ratings. Despite being context-independent traits [13], many studies have shown their impact on economic games (see [8] for a review).

On the other hand, some researchers have introduced other sources of variability between individuals, referred to as narrow traits, to account for specific behaviors observed in classical economic games (Dictator Game, Ultimatum Game, and Prisoner’s Dilemma). These narrow traits include social-value orientation [14], trust tendency [15], and inequality aversion [16], which refers directly to the context of a game. Studies have shown that these narrow traits tend to have better predictive power than broad traits [12, 17, see 18 for a meta-analysis], but they are also more specific and may not be applicable outside of their limited context. This trade-off between broad and narrow traits is known as the bandwidth-fidelity dilemma in the literature [19, 20].

In order to bridge the gap between broad personality traits and game-specific narrow traits, we propose a novel approach that applies the same methodology used for discovering the broad personality traits, i.e a multidimensional analysis of self-ratings [10, 21], within the context of a game. This methodology contrasts with the “contextual personality” approach [22] in social science, because we may observe the emergence of new traits instead of a transposition of a trait from the FFM in a particular context [23, 24]. This approach is expected to yield game-dependent traits that are more generalizable while maintaining a close relationship with behaviors in games. Actually, it has been shown that contextualized traits were more closely associated with contextualized outcomes [25]. In this study, we introduce the game of Bridge as an ideal candidate for this purpose.

**Fig 1:**
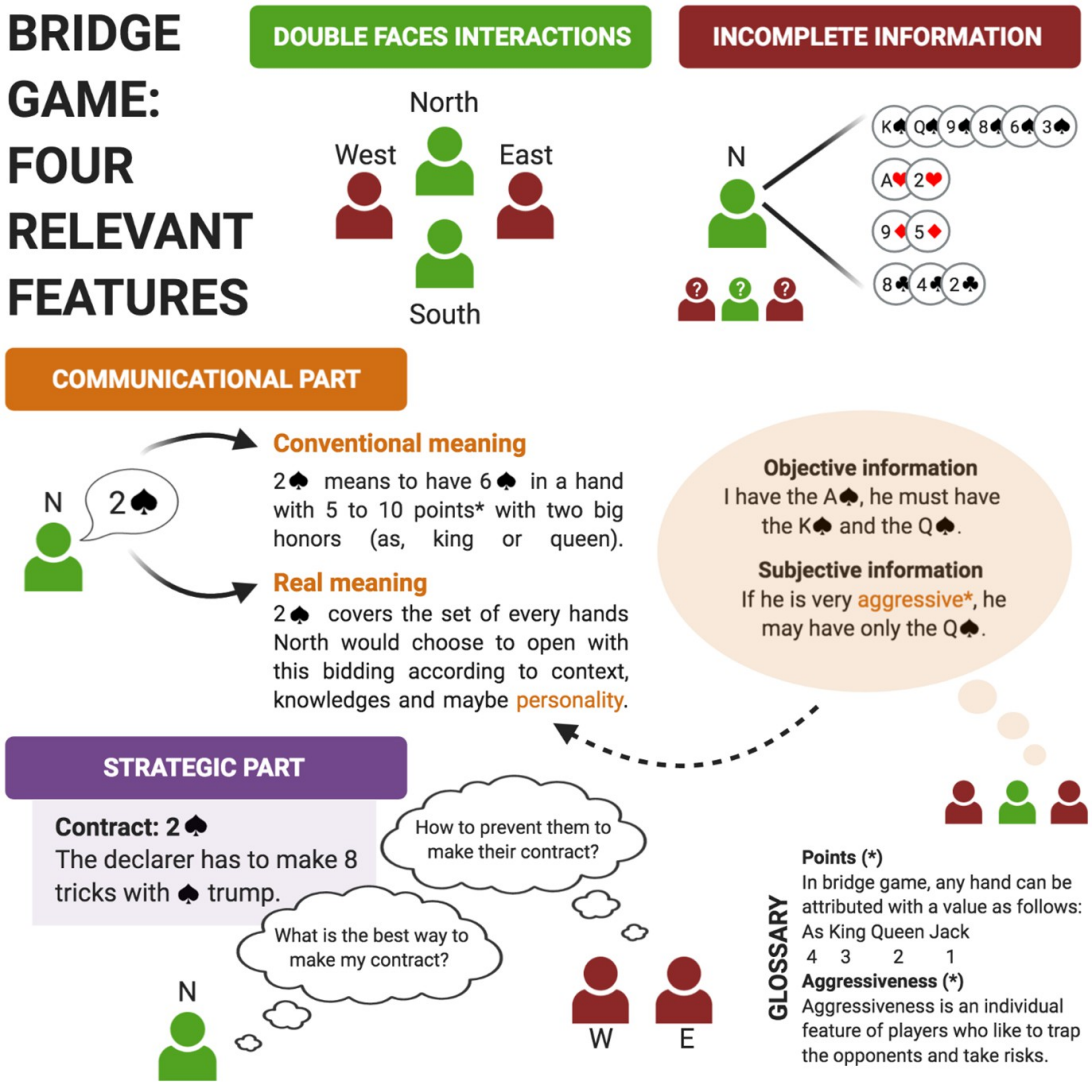
Four characteristics of the game of Bridge. Interactions between players are competitive and cooperative. This is an incomplete information game (players know only their hand) with first, a communicational part (bids implying representations about the real meaning), and then, a strategic part (declarer plays using probabilistic reasoning and inferences).

First and foremost, the game of Bridge is a real-life game where players have already learned the economic as well as social consequences of their choices. This social aspect is often absent in traditional economic games, but it plays a crucial role in real-life social interactions (e.g. [26, 27]) Secondly, the game of Bridge exemplifies a social, cooperative, competitive, and uncertain environment, making it highly reflective of real-life interactions. Indeed, the game of Bridge (Fig 1) is 1- an incomplete information game (as opposed to Chess and Go), 2- a competitive and cooperative game (Chess, Go and Poker are competitive games only), and 3- a game which leaves room to communication between players [28]. Thus, due to its real-life nature and its ability to replicate features of actual social interactions, the game of Bridge is well-suited for revealing a general and coherent structure of personality traits that are relevant for game-like interactions.

Using the game of Bridge as our chosen game allows us to explore a question that has been debated in both the field of personality theory and game theory: the identification of different types of individuals. Traditionally, personality psychology has favored the factorial approach over the typological approach. However, a recent study [29] has partially confirmed the ARC typology [30–32] by utilizing new analysis techniques and large databases. They identified four personality types—the role model type (resilient), the reserved type (overcontrolled), the self-centered type (undercontrolled), and an average type. They suggest that replication issues [11, 33] and the poor predictive validity of previous typologies [34, 35] can be overcome, thanks to the improved methodology.

In the field of behavioral economics, efforts have been made to classify players based on the strategies they use during games, resulting in narrow typologies such as contemplative and impulsive players [36], recursivity reasoning abilities [37], and cooperative, individualistic, and competitive players [38]. Within the video game industry, attempts have also been made to delineate types of video game players [39]. In this study, we propose an intermediate level typology of players based on game-related traits that organize variability among players.

Can we unravel personality traits that are generalizable and transferable, while still being relevant to the context of games? In this study, we employ a data-driven approach to explore game-related traits that capture the inherent variability among players in the game of Bridge. Through our newly created Bridge Inventory, which encompasses a wide collection of descriptors of the game, we have identified five game-related traits and proposed a classification of players into three distinct types. Furthermore, to gain deeper insights into the relationship between these game-related self-rated traits and broad personality traits we compare our findings with the broad traits of the Five-Factor Model [40]. This study sheds light on how different facets of personality manifest in a specific context, and paves the way for further investigations into the interplay between personality and the context of strategic interactions.

## Methods

### Participants

Participants were recruited via specialized websites, social networks and national bridge federations (from June 15th 2022 to September 15th 2022). A total of 1300 bridge players took part in the study. We included in our analysis the complete responses of 815 players. The sample consisted of 533 men (65.4%) and 282 women (34.6%). Among them, 543 participants completed the survey in French and 272 in English, representing 66.6% and 33.3% of the sample, respectively. The mean age of the participants was 58.3 years old (SD = 16.3).

### Survey composition

The survey includes 66 items related to the game of Bridge (1), a short 15-item Big Five Inventory (2), and demographic information (3).

#### Bridge Inventory design

To capture as many aspects of the game of Bridge as possible, we consulted twenty expert players for their opinions on variability between players and the way they may adapt to it. Based on their feedback, we identified several broad categories of potential traits, including players’ reasons for playing, attitudes toward partners and opponents, willingness to explore during the game, reasoning style, reliance on intuition, and creativity. We then designed 66 items for participants to describe themselves on these aspects. The survey includes 55 ordinal variables (Likert scale in 6 points) and 11 categorical variables. (Refer to S1 Table for the full list of the 54 retained items).

**Table:** 

Examples of item:	*Yes*, *definitely*	*Yes*	*Possibly yes*	*Possibly not*	*No*	*Definitely not*
*I’m playing bridge for pleasure*, *I like to spend my free time with other players*.						
*I avoid showing my negative reactions to my partner*.						
*I think in a logical and technical way*.						

#### Short 15-items Big Five Inventory

We utilized the 15-item short version of the Big Five Inventory (BFI), as presented by [40], which exhibits a high mean correlation of 0.9 with the original 44-item BFI scale described by [41]. This abbreviated version was chosen because the Five-Factor Model (FFM) serves as a secondary model of interest for comparison with our newly developed Bridge Inventory. The BFI has been extensively validated, measuring a construct refined over many years of research [42], ensuring the selection of the most effective items for assessment.

#### Demographic information

Participants were also asked to provide their age, sex, and bridge experience, including the number of years of practice, perceived level of expertise, and official ranking.

### Procedure

The survey was made available on SurveyMonkey for a period of two months and was distributed through the French bridge federation, social networks, and websites dedicated to the game of Bridge. Participation was voluntary and unpaid. Participants were informed that their responses would be used for a quantitative and scientific study in cognitive sciences, and that their anonymity would be preserved. After reading the description of our project on bridge players’ "styles," participants could proceed to complete the survey. Consent of participants have been obtained by clickwrap after being informed that the data would be recorded and that the questionnaire was anonymous. The protocol has been reviewed by the ethic committee of the University U-Paris Cité (IRB: 00012022–39).

### Data preprocessing

Items of the Bridge Inventory were normalized and selected to remove near-zero variance variables and eliminate redundancy, following the approach described in [43], in order to ensure the interchangeability of the variables. We, then, removed 11 items where 95% of participants agreed and one item with a correlation of 0.80 with another item. Personality trait scores were also normalized (mean = 0 and standard deviation = 1) in order to remove any selection bias from our dataset as our participant sample is composed of competitive players.

### Statistical analysis

All analyses were performed using R version 3.6.3. Analysis of variances was computed using the "aov" function in R and Fisher tests (F). Student’s t-tests were used to compare two means or test the significance of correlations (Pearson’s r), and were two-sided. Machine learning algorithms are described in S1 Text.

## Results

### Can we uncover game-related traits from our Bridge Inventory?

**Fig 2:**
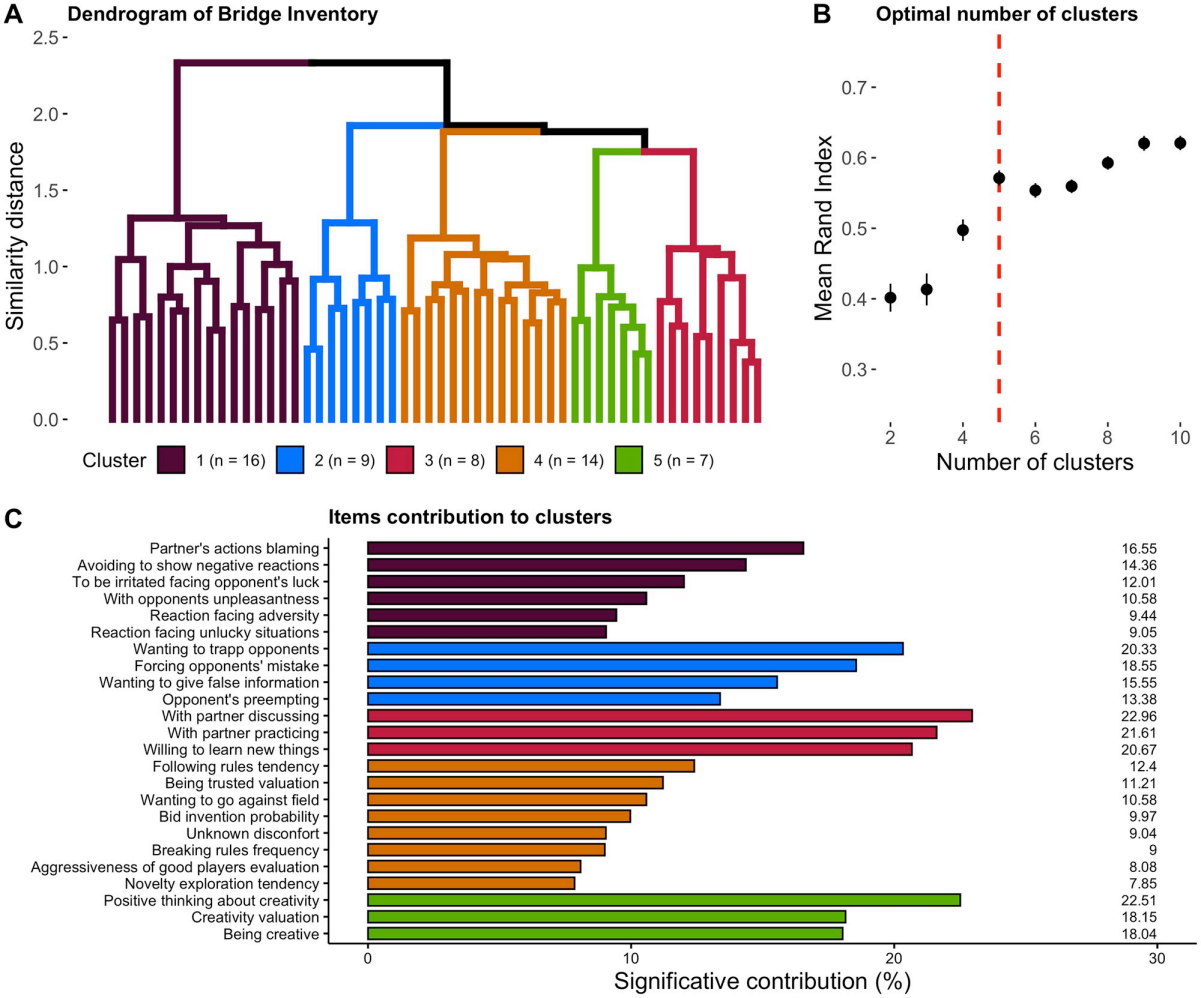
Hierarchical clustering analysis. (A) Dendrogram presenting the results of the hierarchical clustering separated in five clusters including n items. (B) Mean adjusted rand index measuring the similarity between the dendrogram on the left and a dendrogram obtained on hundred bootstraps for every number of clusters from 2 to 10. Error bars show the standard error. (C) Significant contribution of items composing each cluster. The items contributing significantly are the items that contribute more than 100 divided by the number of items included in the cluster (expected contribution under the hypothesis that each item is contributing equally).

We used hierarchical agglomerative clustering for mixed data [44] to summarize the 54 retained Bridge Inventory items. The similarity between agglomerated clusters (Fig 2A) and the stability of the partition (Fig 2B) when compared to hundred bootstraps of the 815 observations indicated that selecting a partition in five clusters was appropriate (ARI = 0.57, SD = 0.11). We performed a principal component analysis on the items within each cluster and based our labeling on the items that had a disproportionate loading on the first principal component (Fig 2C, see S1 Text for the details).

The five resulting clusters were labeled as follows: Cluster 1 (Emotionality) contained items related to emotional and negative reactions of the player; Cluster 2 (Aggressiveness) was centered around the idea of having an active impact on the game and a negative impact on the opponents; Cluster 3 (Experience) reflected the willingness to improve and work on one’s game; Cluster 4 (Discipline) contained items about rules following and conformism; and Cluster 5 (Creativity) was defined by players’ representations about creativity. These clusters represented ecologically relevant and coherent "game-related traits". Henceforth, we used the score on the first principal component as the participant’s score relative to said cluster.

**Fig 3:**
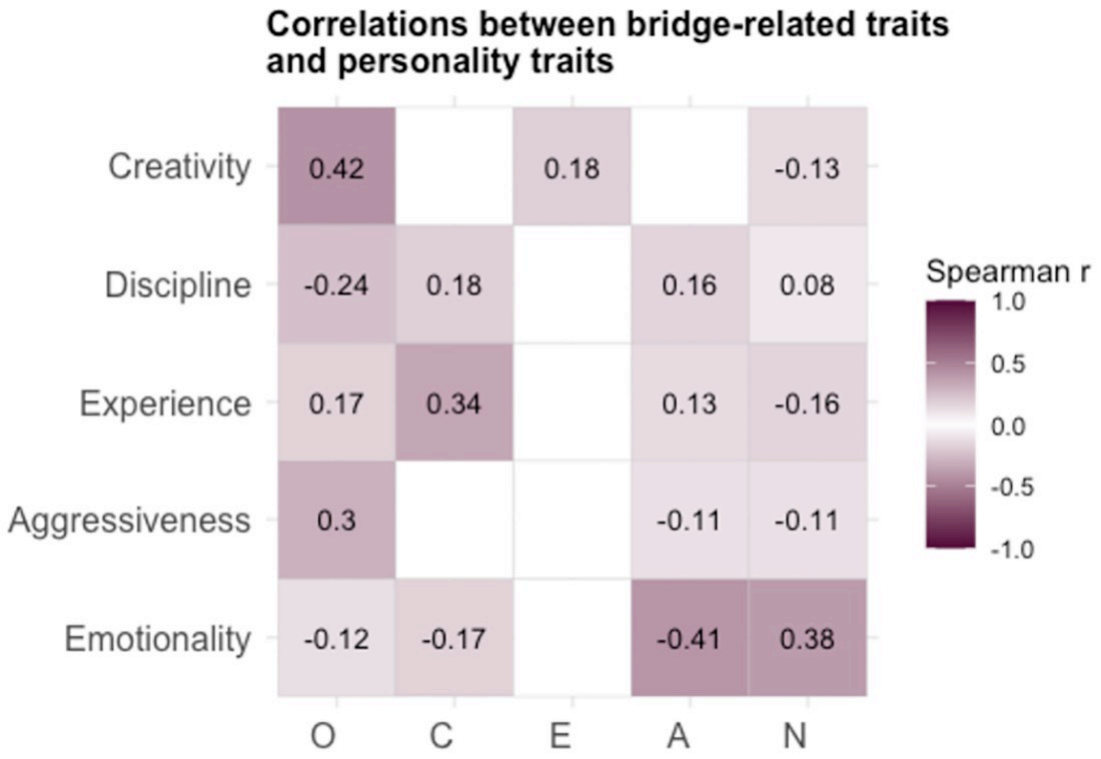
Correlation analysis. Correlation between bridge-related traits and personality traits. Correlation coefficients are Pearson’s r and only significant coefficients are shown. O: Openness, C: Conscientiousness, E: Extraversion, A: Agreeableness, N: Neuroticism.

For further validation of the game-related traits, we examined their relationships with personality traits of the Five-Factor Model (openness, conscientiousness, extraversion, agreeableness, and neuroticism). Correlations between the game-related traits and personality traits are presented in Fig 3. Emotionality was negatively correlated with agreeableness (r = -0.41 [-0.46, -0.35] 95% CI, p < 0.001) and positively correlated with neuroticism (r = 0.38 [0.32, 0.44] 95% CI, p < 0.001). Aggressiveness was correlated with openness (r = 0.30 [0.23, 0.36] 95% CI, p < 0.001), experience with conscientiousness (r = 0.32 [0.28, 0.40] 95% CI, p < 0.001), and creativity with openness (r = 0.42 [0.36, 0.47] 95% CI, p < 0.001).

To enhance the external validity of our Bridge Inventory, we analyzed the effect of demographic variables, after controlling for personality traits (See S2 Table for the full analysis, including demographic variables such as age, gender, and Bridge level, according to the appropriate National Federation). As expected, Aggressiveness is significantly influenced by gender (F(1, 542) = 36.09, p = 0.03, partial η^2^ = 0.95, 95% CI: [0.32, 0.98]). Experience is significantly impacted by both age (F(1,542) = 51.82, p = 0.02, partial η^2^ = 0.96, 95% CI: [0.47, 0.99]) and federal level (F(3,542) = 25.06, p = 0.04, partial η^2^ = 0.97 [0.31, 0.99] 90% CI). Additionally, Discipline shows an increasing trend with age (F(1, 542) = 30.36, p = 0.031, partial η2 = 0.94, 95% CI: [0.24, 0.98]).

### Can we uncover types of players based on our game-related traits?

We utilized a Gaussian Mixture Model (GMM), an unsupervised clustering algorithm, to classify bridge players based on their scores on the five game-related traits. The GMM assumes that the observations belong to k sub-populations or clusters, each represented by a Gaussian component. Further details on the model can be found in S1 Text.

**Fig 4:**
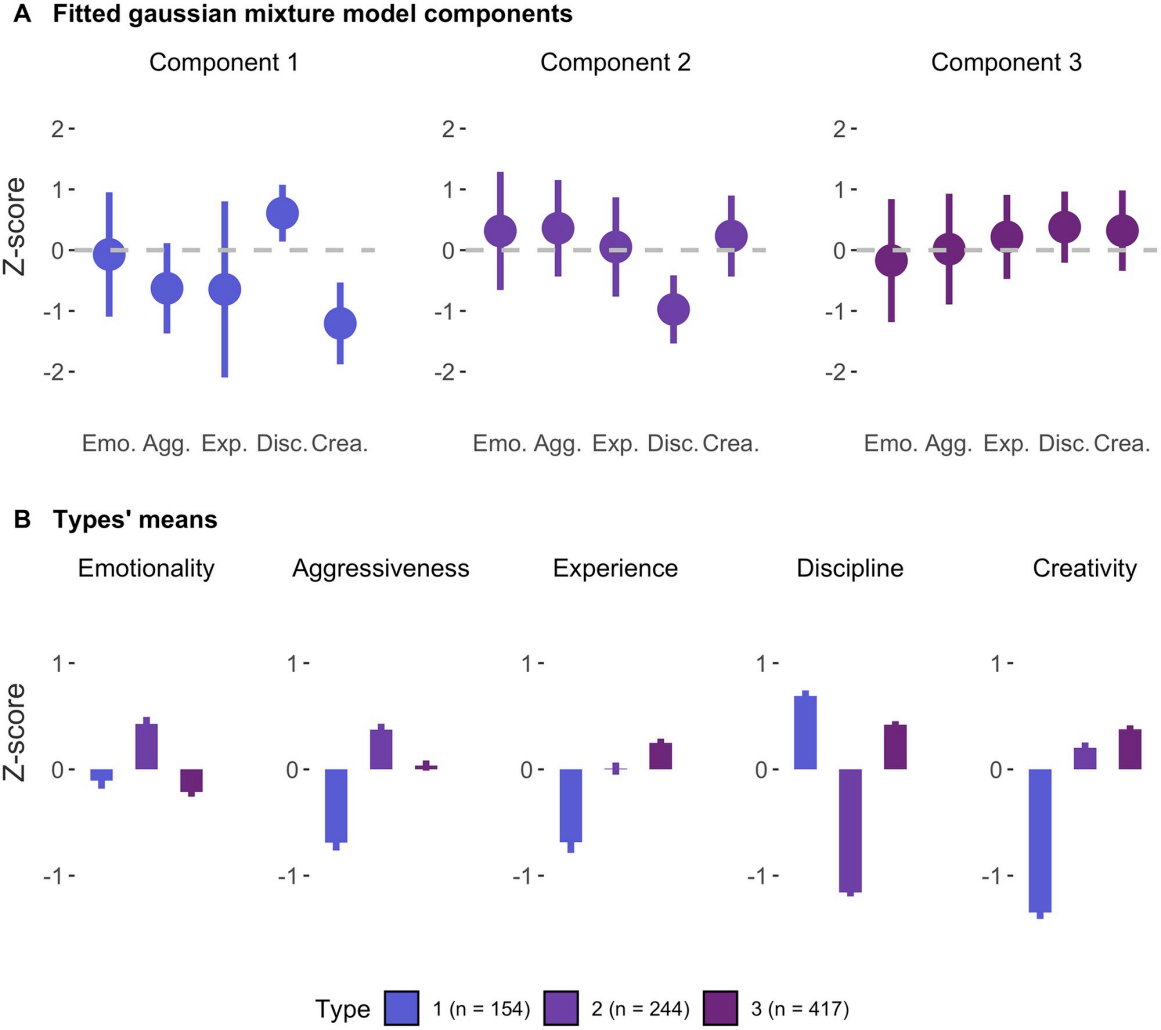
Model fit (A) and participants’ mean scores (B) for the Gaussian Mixture model. (A) Estimated mean z-score of the three gaussian components on each bridge-related trait and standard deviation (error bars). (B) Mean participants’ scores within each type (see S3 Table for descriptive statistics and comparisons with null hypothesis). Error bars represent standard errors; all within traits pairwise comparisons are significant (p < .05 Tukey corrected) except that Type 1 and 3 do not differ on Emotionality (p > .05).

The Likelihood Ratio Test (LRT) indicated a three-groups model based on the comparison models with an increasing k number of components (2 vs 3: LRT = 45.74, p = 0.001; 3 vs 4: LRT = 26.43, p = 0.074). The positions of the three Gaussian components on the game-related traits are depicted in Fig 4A.

Based on the highest posterior probability on the three Gaussian components, players were divided into three types. Fig 4A suggests that the components are primarily dissociated based on discipline and creativity. To further analyze this, we conducted a three-way ANOVA using bridge players’ type as a predictor for each game-related trait and found significant differences for all traits depending on the types (all p < 10^−5^, see S4 Table). Notably, type had a substantial effect on discipline and creativity (η^2^ = 0.58 and η^2^ = 0.43, respectively), while the effect was smaller for emotionality (η^2^ = 0.08), aggressiveness (η^2^ = 0.13), and experience (η^2^ = 0.12).

To further characterize the three types of bridge players, we conducted t-tests with Tukey correction for multiple comparisons to compare the types on each bridge-related trait (Fig 4B and S5 Table). Only one comparison was not significant, with Type 1 and Type 3 showing no significant difference in emotionality (t = 1.14, p = 0.49). In contrast to Types 2 and 3, Type 1 was characterized by low aggressiveness (d = -0.76), experience (d = -0.55), and creativity (d = -1.79), but high discipline (d = 1.09), and thus labeled as "conventional players". Type 2 and Type 3 were more similar to each other, with notable differences in discipline (d = -2.45), but smaller differences in aggressiveness (d = 0.36), experience (d = -0.28), creativity (d = 0.23), and emotionality (d = 0.65). Type 2 was mainly defined by low discipline (d = -1.90) and was labeled as "subversive", while Type 3 showed moderate levels of both creativity (d = 0.62) and discipline (d = 0.52), and was labeled as "measured players".

### Can we recover bridge players’ types from broad personality traits instead of game-related traits?

**Fig 5:**
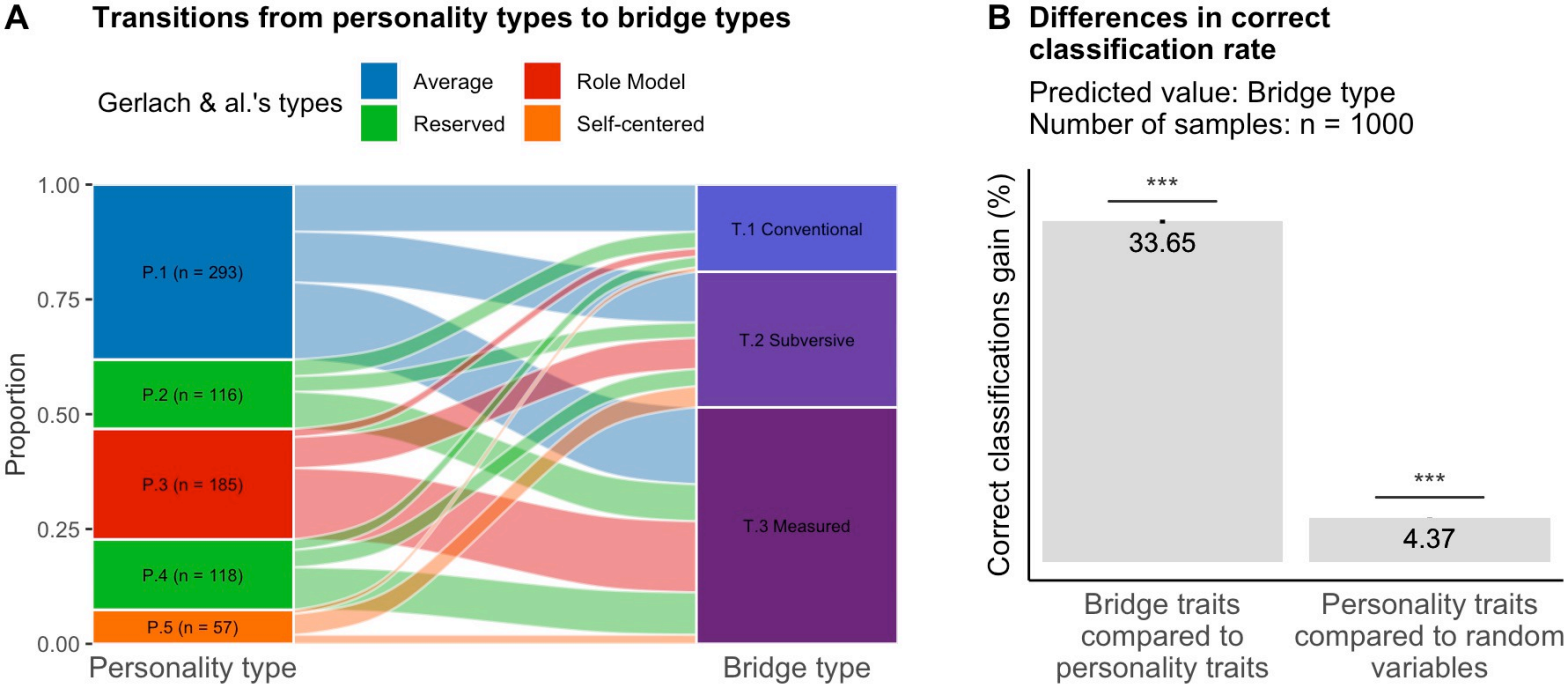
Mutual relationships between the personality-based description and the bridge-related description. (A) Proportion of players of each personality type in the three types of bridge players. Personality types (from P.1 to P.5) represent the five personality types we found whereas colors represent the four Gerlach & al.’s types (see S1 Fig for more details). (B) Differences in correct classification rates using personality traits to predict bridge players’ type compared to the use of bridge traits and random variables. Error bars represent 95% confidence intervals (the right one is too small to be seen).

To investigate this question, we first tested if bridge-related types were contained within personality types. Using a Gaussian mixture model, we classified players based on their personality traits (S1 Fig), and our classification closely matched that in [29], lending support to a comparison with our three bridge types (Fig 5A). Notably, we observed a significant overlap between the two typologies (χ^2^ = 67.02, df = 8, p < 0.0001). We found that the "average" type was over-represented in conventional players (residual value: 0.15), "reserved" and "role model" types were over-classified as measured players (respective residual values: 0.12 and 0.13), and self-centered types were over-classified as subversive players (residual value: 0.31). However, trying to classify players based on the personality type only leads to an accuracy (bridge type recovery rate) of only 44% (theoretical chance: 33.3%).

Despite the fact that the two classifications are not completely aligned, it is still possible that bridge players’ types could be identified using only personality traits, without considering bridge-related traits. To test this hypothesis, we employed a supervised Gaussian mixture model implemented in the R package *mclust*. The model’s performance resulted in 55.6% correct classifications. We evaluated the performance of the model by means of two bootstrap procedures. First, we resampled participants with replacement 1000 times and computed the mean difference in performance for predicting bridge types based on either bridge traits or personality traits. On average, the loss of information incurred by using personality traits instead of bridge-related traits was estimated to be 33.6% (95% CI: [33.4, 33.8]; t = 340.57, p < 0.0001, d = 10.8, 95% CI: [10.3, 11.3]). Next, to estimate the performance expected by chance, we resampled personality traits independently (1000 samples) and tested the performance of a similar supervised model based on these simulated participants. Compared to the bootstrapped performance, actual personality traits resulted in a 4.37% gain in performance (95% CI: [4.35, 4.39]), which was statistically significant (t = 540.06, p < 0.001, d = 17.1, 95% CI: [15.6, 18.8]).

## Discussion

In this article, we take a fresh look at inter-players variability in the game of Bridge. We employ self-ratings of personality trait descriptors and extract dimensional facets of personality that are meaningful in the specific context of this game. With this approach, borrowed from personality theory, we identified five game-related traits that cover crucial aspects of the game: how players react to adversity (Emotionality), their inclination to negatively impact opponents (Aggressiveness), the importance given to the improvement of their abilities (Experience), their reliance on established norms and conformism (Discipline), and their reliance on creativity and intuition (Creativity). Since we used self-ratings to uncover these traits, we suggest that they may be generalizable to any strategic interaction that would share with contract bridge the components of incomplete information, cooperativeness and competitiveness and a definite set of rules. We then defined three player types based on these Bridge-related traits, primarily distinguished by Discipline and Creativity: some players are both conformist and creative (Measured players), while others are biased towards Creativity (Subversive players) or Discipline (Conventional players). Because these types are uncovered based on generalizable game-related traits, they might summarize the major part of variability between players in the game of Bridge.

Our game-related traits reveal novel insights into individual variability that complement and extend the traditional broad personality traits. Particularly, we shed light on the significant role of interpersonal interactions among players, with the Experience trait capturing willingness to improve with one’s partner, Aggressiveness focusing on strategic actions aimed at negatively impacting opponents (such as trapping), and Emotionality reflecting negative reactions towards both partners and opponents. We could then expect these game-related traits to be correlated with Agreeableness and Extraversion, the two broad personality traits which encompass a social dimension [45]. However, Aggressiveness and Experience are not significantly correlated with Extraversion and their correlation with Agreeableness is weak (r = -0.11 and r = 0.13 respectively). On the other hand, Emotionality is weakly correlated with Agreeableness (r = 0.18) but strongly linked to both Agreeableness and Neuroticism (r = -0.41 and r = 0.38 respectively). Thus, our game-related traits of Aggressiveness and Experience reveal new aspects of the interaction between players, while Emotionality captures the unique interplay between Agreeableness and Neuroticism as manifested through our reactions to adversity or difficult situations in the context of a game.

Another noteworthy aspect is the prominence of Discipline, which emerges as a key trait characterizing different types of players, reflecting adherence to rules learned and conformism, as well as the importance of being trusted by one’s partner. Interestingly, Discipline is the only game-related trait that does not reach a correlation of 0.3 with at least one Five Factors Model (FFM) trait, highlighting its specificity to the context of a game, where there are both a set of explicit rules that must be obeyed and a set of recommendations that functions as quasi-rules in standard situations (note, however, that the aspects of unconventionality and rebelliousness are present in the Dutch version of the Openness trait [46]). In the context of cooperative games, this dimension implying being trusted by one’s peers may be essential [47–49], and in competitive games, the ability to regulate impulsive tendencies for the sake of fun or exhibit self-control may be valuable [50].

As concern Creativity, this dimension already appears as a facet of Openness [51] and is positively correlated with Openness (r = 0.42), as expected. It is noteworthy to mention that the item that scores the highest on Creativity is not "to be creative", but "to think that creativity is a valuable characteristic of good players." This suggests that this aspect of Creativity, as measured by our Bridge Inventory, may be used as a strategic approach rather than an inherent characteristic of individuals.

While the content of our game-related traits is novel and relevant for strategic interactions, it exhibits strong correlations with known FFM traits, with the exception of Discipline. These correlations fell within the range of personality facets with FFM traits [52] highlighting the close link between the two taxonomies. As recommended in [53], we can interpret our game-related traits as facets of FFM traits, while maintaining their specificity. Indeed, we may interpret Emotionality as the interindividual aspect in the interaction between Agreeableness and Neuroticism, Experience as the cooperative aspect of Conscientiousness, Aggressiveness as an adversarial aspect of Openness, and Creativity as a strategic aspect of Openness. Besides, our game-related traits, as FFM traits, are influenced by factors as gender [54], age [55] or even experience, as is found in the field of sport psychology [56].

The interpretation of the five game-related traits revealed in our study as facets of personality can be understood in two ways: either game-related traits are novel facets of personality that only manifest in the context of a game, or game-related traits are the translation of pre-existing facets of personality into the context of the game of Bridge. The first hypothesis aligns with the classical view of personality as a hierarchical model, where broad dimensions of personality are linked to specific behaviors [51], including contextual facets. On the other hand, the second hypothesis is in line with the Cognitive-Affective Personality System (CAPS) proposed in [57], which suggests that personality states can change depending on the situation. However, as players in our study completed both the Inventories together, with the Big Five Inventory following the Bridge Inventory, the context of the game of Bridge was present throughout the assessment. Yet, we still observed significant differences in the traits revealed.

Our game-related traits led us to identify three distinct types of players, which reflect unique combinations of traits not captured by classical personality models based on broad personality traits at either the typological or trait level. At the typological level, comparing these player types to those defined in [29], we found that conventional players were more likely to fall into the "average personality" type, while "role-model" and "reserved" types were more likely to be classified as measured players, and "self-centered" types as subversive players. However, these classifications were imperfectly aligned as they matched for only 44% of the players. At the trait level, we discovered that using FFM traits to classify players in the three bridge player types resulted in a 34% decrease in the correct classification rate compared to using game-related traits. This difference suggests that game-related traits contain unique information beyond broad personality traits, allowing for an original description of between-player variability in the game of Bridge.

## Conclusions

Our study has revealed a unique space of game-related traits and types that are not only generalizable but also shed a new light on the interindividual variability among players. We believe that this intermediate level description of players provides valuable insights into the complexity of the interaction between broad personality traits and specific contexts where behaviors are observed. With our study, we hope to provide new knowledge about interindividual variability in the context of social interactions and to open up a new path for researchers to explore the hidden layers of personality traits that lie beyond the dichotomy between broad and narrow.

## Supporting information

S1 Text Supplementary methods. Machine learning algorithm are described.
(DOCX)

S1 Table Bridge Inventory items. 54 items retained after removing near-0 variance variables and closely related items.
(DOCX)

S2 Table Analysis of variance of bridge-related traits depending on personality traits, demographic information and practice of bridge game. (DOCX)

S3 Table Descriptive statistics of the three bridge players’ types and one-sample t-tests. (DOCX)

S4 Table Effect of bridge players’ type on each bridge-related trait. F-statistics have been computed thanks to a one-way ANOVA.
(DOCX)

S5 Table Multiple comparison of bridge players’ types on each bridge-related trait. Multiple comparisons have been performed using a t-test corrected with a Tukey method for comparing three groups (T.1: conventional players, T.2: subversive players, T.3: measured players).
(DOCX)

S1 Fig Gaussian mixture components on personality traits compared to types founded by Gerlach & al. (2018). We select seven clusters thanks to the Likelihood Ratio Test (LRT = 16.39, p = 0.157) and choose to remove two clusters that represent less than 5% of bridge players (1.59% and 4.05%). Each of the five retained component is presented with its mean and standard deviation. Gerlach & al.’s types have been graphically estimated. Components are presented with the closest (minimum euclidean distance) Gerlach & al.’s type. The five personality traits are O: Openness, C: Conscientiousness, E: Extraversion, A: Agreeableness and N: Neuroticism.
(TIF)
