# Supplementary material for: Beyond broad and narrow: Intermediate level traits in the personality of bridge players
Source: PLoS One. 2024 Aug 22;19(8):e0305985. doi: 10.1371/journal.pone.0305985 (PMC11340889; doi:10.1371/journal.pone.0305985)
Supplement: S1 Text — Machine learning algorithm are described. (DOCX) [file pone.0305985.s001.docx]

Beyond Broad and Narrow: Intermediate level traits in the Personality of Bridge players

**Camille Sauvain, Véronique Ventos & Jérôme Sackur**

# Supplementary methods

**Hierarchical clustering.**

Hierarchical clustering is a clustering algorithm where, at each step, the two closest variables are grouped, forming a new variable, until there remains only one variable. The similarity between variables is based on the homogeneity $H$of a group of variables $C_{k}$ such as :

$$H(C_{k}) =\sum_{x \in C_{k}} r_{x, c_{k}}^{2}+ \sum_{y \in C_{k}} \eta_{c_{k}|y}^{2}$$

$r_{x, c_{k}}^{2}$is the squared Pearson correlation between quantitative variables$x$ in $C_{k}$and $c_{k}$ and $\eta_{c_{k}|y}^{2}$is the correlation ratio between qualitative variables$y$in $C_{k}$and $c_{k}$ measuring the part of variance of $c_{k}$explained by the categories of$y.$

$c_{k}$ is the synthetic variable defined as the quantitative variable the “most linked” to all the variables in $C_{k}$:

$$c_{k} ={arg max}_{u \in R^{n}} (\sum_{x \in C_{k}} r_{x, u}^{2}+ \sum_{y \in C_{k}} \eta_{u|y}^{2} )$$

$c_{k}$ correspond to the first principal component of a PCA applied to the set of items contained in the cluster k.

This algorithm is implemented in the R package ClustOfVar (Chavent & al., 2012). To select the number of clusters we use the distance between aggregated clusters and the adjusted rand index (Rand, 1971) computed between our partition and partitions obtained using 1000 bootstraps.

The adjusted rand index (ARI) quantifies the link between two classifications and is corrected for chance (Rand, 1971).

| Contingency table | | **Classification 1** | |
| --- | --- | --- | --- |
|  |  | *Category 1* | *Category 2* |
| **Classification 2** | *Category 1* | a | b |
|  | *Category 2* | c | d |

$$ARI = \frac{2(ad + bc)}{(a+b)(b+d) + (a+c) (c+d)}$$

**Gaussian mixture model.**

A gaussian mixture model assumes the$n$ observations of$\boldsymbol{x}$ to be drawn from a mixture of $G$ Gaussian distributions named components, each one corresponding to one cluster. The probability density function is defined as :

$f(x_{i}, \Psi) =\sum_{k = 1}^{G} \pi_{k}N{(\mu}_{k},\Sigma_{k})$

$\Psi$ are the parameters of the model: $\pi_{k}$the mixing weights, $\mu_{k}$the mean vector and $\Sigma_{k}$the covariance matrix. Parameters are fitted thanks to an expectation-maximization algorithm implemented in the R package *mclust* (Scrucca & al., 2016). To select the number of clusters we use the Likelihood ratio test and we compare the density of the center of our clusters with the density at the same location in 10.000 randomized data sets.

The Likelihood ratio test is an incremental technique used to decide if choosing a partition in k+1 clusters is better than choosing a partition in k clusters. ${\hat{\Psi}(k)}$is the maximum likelihood estimate of $\Psi$ for a partition in k clusters. The LRT compares it to the likelihood estimate of $\Psi$for a partition in k+1 clusters such as:

$LRT = 2[L(\hat{\Psi}(k+1)) - L(\hat{\Psi}(k))]$ where $L$ is the log-likelihood function.

We compare this observed LRT (${LRT}_{obs})$ with the LRT on 1000 ($B$) bootstraps (${LRT}_{b})$ to approximate the null distribution and compute the p value:

$p\approx\frac{1 + \sum_{i = 1}^{B} I({LRTS}_{b} \geq{LRTS}_{obs})}{B+1}$ where $I(.)$ is an indicator function which is equal to 1 if ${LRT}_{b} \geq{LRT}_{obs}$and 0 otherwise (Scrucca & al., 2016).

To estimate the significance of the density at the center of the clusters, we draw 10.000 randomized data sets shuffling our data in each trait (bridge traits or personality traits). We perform a GMM on each randomized data set allowing us to obtain the distribution of the density at each point of the space. The density observed at the center of our clusters is compared to this distribution at the same location in the 10.000 randomized data sets. We can quantify the differences in density by $\frac{\rho}{\rho^{*}}$ where $\rho$ is the observed density and $\rho^{*}$the mean of the simulated distribution. This analysis replicates Gerlach & al. (2018).
