# Supplementary material for: Beyond broad and narrow: Intermediate level traits in the personality of bridge players
Source: PLoS One. 2024 Aug 22;19(8):e0305985. doi: 10.1371/journal.pone.0305985 (PMC11340889; doi:10.1371/journal.pone.0305985)
Supplement: S1 Table — 54 items retained after removing near-0 variance variables and closely related items. (DOCX) [file pone.0305985.s002.docx]

Beyond Broad and Narrow: Intermediate level traits in the Personality of Bridge players

**Camille Sauvain, Véronique Ventos & Jérôme Sackur**

## Bridge Inventory Items

**S1 Table. Bridge Inventory Items.** 54 items retained after removing near-0 variance variables and closely related items.

| **Question** | **Response form** |
| --- | --- |
| I'm playing bridge for the pleasure, I like to spend my free time with other players | Likert scale (6 pts) |
| I'm playing bridge for the glory, I like to find the only lead to beat the contract while others choose a more classic lead | Likert scale (6 pts) |
| I'm playing bridge to win, every (allowed) move is good if it makes me win | Likert scale (6 pts) |
| I'm playing bridge to learn, I like to experiment with new conventions and discuss best plays | Likert scale (6 pts) |
| I'm playing bridge to teach, I like to help others and to guide new players to improve themselves | Likert scale (6 pts) |
| When I choose a bid, my goal is reaching a good contract as rapidly as possible | Likert scale (6 pts) |
| When I chose a bid, my goal is giving a false information to opponents | Likert scale (6 pts) |
| When I chose a bid, my goal is trapping opponents | Likert scale (6 pts) |
| When I chose a bid, my goal is making opponents make the last guess | Likert scale (6 pts) |
| I think that most of players would provide the same answer as me to the two last questions (relative to motivations) | Likert scale (6 pts) |
| I might blame the actions of my partner | Likert scale (6 pts) |
| I often discuss plays/situations with my partner | Likert scale (6 pts) |
| I avoid showing my negative reactions to my partner | Likert scale (6 pts) |
| I often congratulate my partner (even if not totally justified) | Likert scale (6 pts) |
| I try to justify my decisions | Likert scale (6 pts) |
| I try to practice with my partner | Likert scale (6 pts) |
| I support my partner's decisions even if I think it was not the best play | Likert scale (6 pts) |
| I often joke with my opponents | Likert scale (6 pts) |
| I am as neutral as possible with my opponents | Likert scale (6 pts) |
| It amuses me when my opponents make a mistake | Likert scale (6 pts) |
| I try to preempt opponents in order to have them make mistakes | Likert scale (6 pts) |
| I'm irritated when my opponents are lucky | Likert scale (6 pts) |
| I don't like it when my opponents are playing "exotic" conventions | Likert scale (6 pts) |
| I can be unpleasant with my opponents | Likert scale (6 pts) |
| I think that good players try to force opponents into making a mistake | Likert scale (6 pts) |
| I think that good players don't pay attention to the impact of their actions on their opponents | Likert scale (6 pts) |
| I think that good players must be creative | Likert scale (6 pts) |
| I think it is better to be trusted by partners than to let run my imagination | Likert scale (6 pts) |
| I think you need to be able to follow rules to be a good player | Likert scale (6 pts) |
| I take into account opponent's creativity when I'm playing in a competition | Likert scale (6 pts) |
| I think I am creative | Likert scale (6 pts) |
| I often break rules of bidding | Likert scale (6 pts) |
| Being creative is an advantage | Likert scale (6 pts) |
| I make decisions rapidly without thinking about pros and cons | Likert scale (6 pts) |
| I can declare a contract without knowing why or having good reason to do so | Likert scale (6 pts) |
| I rather think globally than making a precise estimation of likelihoods | Likert scale (6 pts) |
| I think you need intuition to be a good player | Likert scale (6 pts) |
| I think in a logical and technical way | Likert scale (6 pts) |
| I like to go against the field in pairs tournaments | Likert scale (6 pts) |
| I think a good player is often going against the field in pairs tournament | Likert scale (6 pts) |
| I think that most of players would provide the same answer as me to the two last questions (relative to emotionality) | Likert scale (6 pts) |
| I think good players are very aggressive | Likert scale (6 pts) |
| I think you need to be aggressive to be a good player | Likert scale (6 pts) |
| I take into account the style of my opponents when I am playing | Likert scale (6 pts) |
| What is your greater strength | Likert scale (6 pts) |
| I play other games | Binary scale |
| I have ever made up a bid | Binary scale |
| I know someone who is creative | Binary scale |
| I like to test new conventions during competitions | Binary scale |
| I have ever made I bid without knowing if my partner would correctly interpret it | Binary scale |
| Scoring a 0 in pair tournament   1. Something which happens but I try to avoid it 2. Not a big deal if I’m scoring more tops than bottoms 3. Not a big deal if I think the bid was good or the line of play was the best 4. A persistent fear, that’s why I stick with the field 5. Not a big matter if it was fun | QCM |
| Your partner issues a game try in a pairs tournament and you have a borderline hand   1. You decline, cards are always ill placed 2. You accept, an opponent may be willing to give me a trick 3. You accept, I am very good as declarer 4. You decline, I don’t like to play difficult contracts 5. You seek any little reason to accept 6. You seek any little reason to refuse | QCM |
| When you score a 0   1. You are angry, this field is really bad 2. You blame your partner for making a mistake 3. You already think to the next tricks you could use to make up for this bad score 4. You will make your opponents pay for it 5. You keep calm, there are many deals left to play 6. You are disappointed by yourself | QCM |
| When a tournament starts badly   1. You feel down 2. You go against the field to compensate 3. You let things cool down 4. You begin to be annoyed at your partner 5. You begin to be angry at your opponents 6. You try to focus, as much as possible, on the next deals | QCM |
