# Supplementary material for: Beyond broad and narrow: Intermediate level traits in the personality of bridge players
Source: PLoS One. 2024 Aug 22;19(8):e0305985. doi: 10.1371/journal.pone.0305985 (PMC11340889; doi:10.1371/journal.pone.0305985)
Supplement: S2 Table — (DOCX) [file pone.0305985.s003.docx]

Beyond Broad and Narrow: Intermediate level traits in the Personality of Bridge players

**Camille Sauvain, Véronique Ventos & Jérôme Sackur**

# Supplementary results

| **S2 Table. Analysis of variance of bridge-related traits depending on personality traits, demographic information and practice of bridge game.** | | | | | | | |
| --- | --- | --- | --- | --- | --- | --- | --- |
| **Dependant Variable** | **Independent Variables** | | **df** | **F** | **p** | **Partial**  **𝝶^2^** | **90% CI Partial 𝝶^2^** |
| Emotionality | Personality traits | Openness | 1, 542 | 1.73 | 0.3193 | 0.46 | 0.00, 0.83 |
|  |  | Conscientiousness | 1, 542 | 3.31 | 0.2103 | 0.62 | 0.00, 0.88 |
|  |  | Extraversion | 1, 542 | 0.81 | 0.4622 | 0.29 | 0.00, 0.77 |
|  |  | Agreeableness | **1, 542** | **87.36** | **0.0113** | **0.98** | **0.64, 0.99** |
|  |  | Neuroticism | **1, 542** | **77.19** | **0.0127** | **0.97** | **0.60, 0.99** |
|  | Demographic variables | Age | 1, 542 | 7.82 | 0.1076 | 0.80 | 0.00, 0.93 |
|  |  | Gender | 1, 542 | 0.24 | 0.6750 | 0.11 | 0.00, 0.68 |
|  | Bridge-related variables | Federal level | 3, 542 | 6.44 | 0.1373 | 0.91 | 0.00, 0.97 |
|  |  | Perceived level | 3, 542 | 3.89 | 0.2111 | 0.85 | 0.00, 0.95 |
|  |  | Years of practice | 1, 542 | 0.07 | 0.8188 | 0.03 | 0.00, 0.57 |
| Aggressiveness | Personality traits | Openness | **1, 542** | **56.77** | **0.02** | **0.97** | **0.50, 0.99** |
|  |  | Conscientiousness | 1, 542 | 0.16 | 0.73 | 0.07 | 0.00, 0.65 |
|  |  | Extraversion | 1, 542 | 0.09 | 0.79 | 0.04 | 0.00, 0.60 |
|  |  | Agreeableness | 1, 542 | 6.70 | 0.12 | 0.77 | 0.00, 0.93 |
|  |  | Neuroticism | 1, 542 | 10.21 | 0.09 | 0.84 | 0.00, 0.95 |
|  | Demographic variables | Age | 1, 542 | 1.41 | 0.36 | 0.41 | 0.00, 0.81 |
|  |  | Gender | **1, 542** | **36.09** | **0.03** | **0.95** | **0.32, 0.98** |
|  | Bridge-related variables | Federal level | 3, 542 | 12.62 | 0.07 | 0.95 | 0.00, 0.98 |
|  |  | Perceived level | 3, 542 | 3.11 | 0.25 | 0.82 | 0.00, 0.91 |
|  |  | Years of practice | 1, 542 | 4.69 | 0.16 | 0.70 | 0.00, 0.90 |
| Experience | Personality traits | Openness | **1, 542** | **24.86** | **0.04** | **0.93** | **0.14, 0.98** |
|  |  | Conscientiousness | **1, 542** | **327.90** | **0.003** | **0.99** | **0.89, 1.00** |
|  |  | Extraversion | 1, 542 | 17.93 | 0.051 | 0.90 | 0.00, 0.95 |
|  |  | Agreeableness | 1, 542 | 16.44 | 0.06 | 0.89 | 0.00, 0.96 |
|  |  | Neuroticism | **1, 542** | **20.12** | **0.05** | **0.91** | **0.04, 0.97** |
|  | Demographicvariables | Age | **1, 542** | **51.82** | **0.02** | **0.96** | **0.47, 0.99** |
|  |  | Gender | 1, 542 | 0.94 | 0.43 | 0.32 | 0.00, 0.78 |
|  | Bridge-related variables | Federal level | **3, 542** | **25.06** | **0.04** | **0.97** | **0.31, 0.99** |
|  |  | Perceived level | 3, 542 | 7.56 | 0.12 | 0.92 | 0.00, 0.97 |
|  |  | Years of practice | 1, 542 | 0.31 | 0.64 | 0.13 | 0.00, 0.70 |
| Discipline | Personality traits | Openness | 1, 542 | 9.62 | 0.090 | 0.83 | 0.00, 0.94 |
|  |  | Conscientiousness | 1, 542 | 5.70 | 0.140 | 0.74 | 0.00, 0.92 |
|  |  | Extraversion | 1, 542 | 0.19 | 0.702 | 0.09 | 0.00, 0.66 |
|  |  | Agreeableness | 1, 542 | 2.35 | 0.265 | 0.54 | 0.00, 0.85 |
|  |  | Neuroticism | 1, 542 | 3.22 | 0.215 | 0.62 | 0.00, 0.88 |
|  | Demographic variables | Age | **1, 542** | **30.36** | **0.031** | **0.94** | **0.24, 0.98** |
|  |  | Gender | 1, 542 | 7.08 | 0.117 | 0.78 | 0.00, 0.93 |
|  | Bridge-related variables | Federal level | 3, 542 | 5.68 | 0.153 | 0.89 | 0.00, 0.95 |
|  |  | Perceived level | 3, 542 | 1.12 | 0.504 | 0.63 | 0.00, 0.86 |
|  |  | Years of practice | 1, 542 | 2.68 | 0.243 | 0.57 | 0.0, 0.86 |
| Creativity | Personality traits | Openness | **1, 542** | **51.71** | **0.019** | **0.96** | **0.46, 0.99** |
|  |  | Conscientiousness | 1, 542 | 0.17 | 0.720 | 0.08 | 0.00, 0.65 |
|  |  | Extraversion | 1, 542 | 5.62 | 0.141 | 0.74 | 0.00, 0.92 |
|  |  | Agreeableness | 1, 542 | 0.35 | 0.614 | 0.15 | 0.00, 0.71 |
|  |  | Neuroticism | 1, 542 | 3.91 | 0.186 | 0.66 | 0.00, 0.89 |
|  | Demographic variables | Age | 1, 542 | 1.22 | 0.384 | 0.38 | 0.00, 0.80 |
|  |  | Gender | 1, 542 | 4.90 | 0.157 | 0.71 | 0.00, 0.91 |
|  | Bridge-related variables | Federal level | 3, 542 | 9.22 | 0.099 | 0.93 | 0.00, 0.98 |
|  |  | Perceived level | 3, 542 | 1.59 | 0.409 | 0.70 | 0.00, 0.89 |
|  |  | Years of practice | 1, 542 | 5.54 | 0.143 | 0.73 | 0.00, 0.91 |
