# Supplementary material for: Beyond broad and narrow: Intermediate level traits in the personality of bridge players
Source: PLoS One. 2024 Aug 22;19(8):e0305985. doi: 10.1371/journal.pone.0305985 (PMC11340889; doi:10.1371/journal.pone.0305985)
Supplement: S3 Table — (DOCX) [file pone.0305985.s004.docx]

Beyond Broad and Narrow: Intermediate level traits in the Personality of Bridge players

**Camille Sauvain, Véronique Ventos & Jérôme Sackur**

# Supplementary results

| **S3 Table. Descriptive statistics of the three bridge players’ types and one-sample t-tests.** | | | | | |
| --- | --- | --- | --- | --- | --- |
| **Dependant Variable** | **Type 1 (n = 154)** | | | | |
|  | **Mean** | **SD** | **t** | **p** | **Cohen d**  **(95% CI)** |
| Emotionality | -0.11 | 0.92 | -1.45 | 0.15 | -0.12 (-0.28, 0.04) |
| Aggressiveness | -0.69 | 0.91 | -9.42 | < 10^-5^ | -0.76 (-0.94, -0.6) |
| Experience | -0.68 | 1.25 | -6.77 | < 10^-5^ | -0.55 (-0.72, -0.38) |
| Discipline | 0.69 | 0.63 | 13.58 | < 10^-5^ | 1.09 (0.9, 1.3) |
| Creativity | -1.35 | 0.75 | -22.70 | < 10^-5^ | -1.79 (-2.12, -1.53) |
| **Dependant Variable** | **Type 2 (n = 244)** | | | | |
|  | **Mean** | **SD** | **t** | **p** | **Cohen d**  **(95% CI)** |
| Emotionality | 0.43 | 1.01 | 6.62 | < 10^-5^ | 0.42 (0.3, 0.56) |
| Aggressiveness | 0.37 | 0.87 | 6.74 | < 10^-5^ | 0.43 (0.2, 0.57) |
| Experience | 0.01 | 0.90 | 0.13 | 0.90 | 0.01 (-0.12, 0.13) |
| Discipline | -1.16 | 0.61 | -29.65 | < 10^-5^ | -1.90 (-2.06, -1.76) |
| Creativity | 0.20 | 0.80 | 3.93 | < 10^-5^ | 0.25 (0.12, 0.38) |
| **Dependant Variable** | **Type 3 (n = 417)** | | | | |
|  | **Mean** | **SD** | **t** | **p** | **Cohen d**  **(95% CI)** |
| Emotionality | -0.21 | 0.94 | -4.56 | 0.15 | -0.22 (-0.32, -0.13) |
| Aggressiveness | 0.04 | 0.98 | -0.75 | 0.45 | 0.04 (-0.06, 0.14) |
| Experience | 0.25 | 0.82 | 6.20 | < 10^-5^ | 0.30 (0.21, 0.41) |
| Discipline | 0.42 | 0.66 | 12.73 | < 10^-5^ | 0.62 (0.52, 0.73) |
| Creativity | 0.38 | 0.73 | 10.58 | < 10^-5^ | 0.52 (0.42, 0.62) |
| Note: t values have been computed using a two-sided one-sample t-test. | | | | | |
