# Supplementary material for: Beyond broad and narrow: Intermediate level traits in the personality of bridge players
Source: PLoS One. 2024 Aug 22;19(8):e0305985. doi: 10.1371/journal.pone.0305985 (PMC11340889; doi:10.1371/journal.pone.0305985)
Supplement: S4 Table — F-statistics have been computed thanks to a one-way ANOVA. (DOCX) [file pone.0305985.s005.docx]

Beyond Broad and Narrow: Intermediate level traits in the Personality of Bridge players

**Camille Sauvain, Véronique Ventos & Jérôme Sackur**

| **S4 Table. Effect of bridge players’ type on each bridge-related trait** | | | | |
| --- | --- | --- | --- | --- |
| **Independent variable: Bridge-related traits** | **Main effect of bridge players’ type** | | | |
|  | **F** | **p** | **Partial 𝝶^2^** | **90% CI**  **Partial 𝝶^2^** |
| Emotionality | 35.28 | <10^-5^ | 0.08 | 0.05, 0.11 |
| Aggressiveness | 61.98 | <10^-5^ | 0.13 | 0.10, 0.19 |
| Experience | 55.52 | <10^-5^ | 0.12 | 0.09, 0.15 |
| Discipline | 564.2 | <10^-5^ | 0.58 | 0.55, 0.66 |
| Creativity | 304.13 | <10^-5^ | 0.43 | 0.39, 0.46 |
| Note: F-statistics have been computed thanks to a one-way ANOVA | | | | |
