# Supplementary material for: Beyond broad and narrow: Intermediate level traits in the personality of bridge players
Source: PLoS One. 2024 Aug 22;19(8):e0305985. doi: 10.1371/journal.pone.0305985 (PMC11340889; doi:10.1371/journal.pone.0305985)
Supplement: S5 Table — Multiple comparisons have been performed using a t-test corrected with a Tukey method for comparing three groups (T.1: conventional players, T.2: subversive players, T.3: measured players). (DOCX) [file pone.0305985.s006.docx]

Beyond Broad and Narrow: Intermediate level traits in the Personality of Bridge players

**Camille Sauvain, Véronique Ventos & Jérôme Sackur**

| **S5 Table. Multiple comparison of bridge players’ types on each bridge-related trait** | | | | | | | | | |
| --- | --- | --- | --- | --- | --- | --- | --- | --- | --- |
| **Independent variable: Bridge-related traits** | **Mean comparison between types** | | | | | | | | |
|  | **T.1 vs T.2** | | | **T.1 vs T.3** | | | **T.2 vs T.3** | | |
|  | **t** | **p** | **Cohen d**  **(CI 95%)** | **t** | **p** | **Cohend**  **(CI 95%)** | **t** | **p** | **Cohend**  **(CI 95%)** |
| Emotionality | -5.42 | <10^-5^ | -0.55  (-0.78, -0.37) | 1.14 | 0.49 | 0.11  (-0.09, 0.31) | 8.26 | <10^-5^ | 0.65  (0.48, 0.83) |
| Aggressiveness | -11.08 | <10^-5^ | -1.20  (-1.42, -0.99) | -8.25 | <10^-5^ | -0.77  (-0.96, -0.59) | 4.49 | <10^-5^ | 0.36  (0.21, 0.52) |
| Experience | -7.16 | <10^-5^ | -0.63  (-0.87, -0.42) | -10.54 | <10^-5^ | -0.88  (-1.11, -0.66) | -3.19 | <10^-5^ | -0.28  (-0.44, -0.12) |
| Discipline | 27.71 | <10^-5^ | 2.98  (2.73, 3.27) | 4.44 | <10^-5^ | 0.41  (0.22, 0.6) | -30.2 | <10^-5^ | -2.45  (-2.64, -2.3) |
| Creativity | -19.86 | <10^-5^ | -1.99  (-2.27, -1.75) | -24.16 | <10^-5^ | -2.32  (-2.58, -2.08) | -2.9 | 0.01 | -0.23  (-0.4, -0.08) |
| Note: Multiple comparisons have been performed using a t-test corrected with a Tukey method for comparing three groups (T.1: conventional players, T.2: subversive players, T.3: measured players). | | | | | | | | | |
