# Supplementary figures and images for: Beyond broad and narrow: Intermediate level traits in the personality of bridge players
Source: PLoS One. 2024 Aug 22;19(8):e0305985. doi: 10.1371/journal.pone.0305985 (PMC11340889; doi:10.1371/journal.pone.0305985)

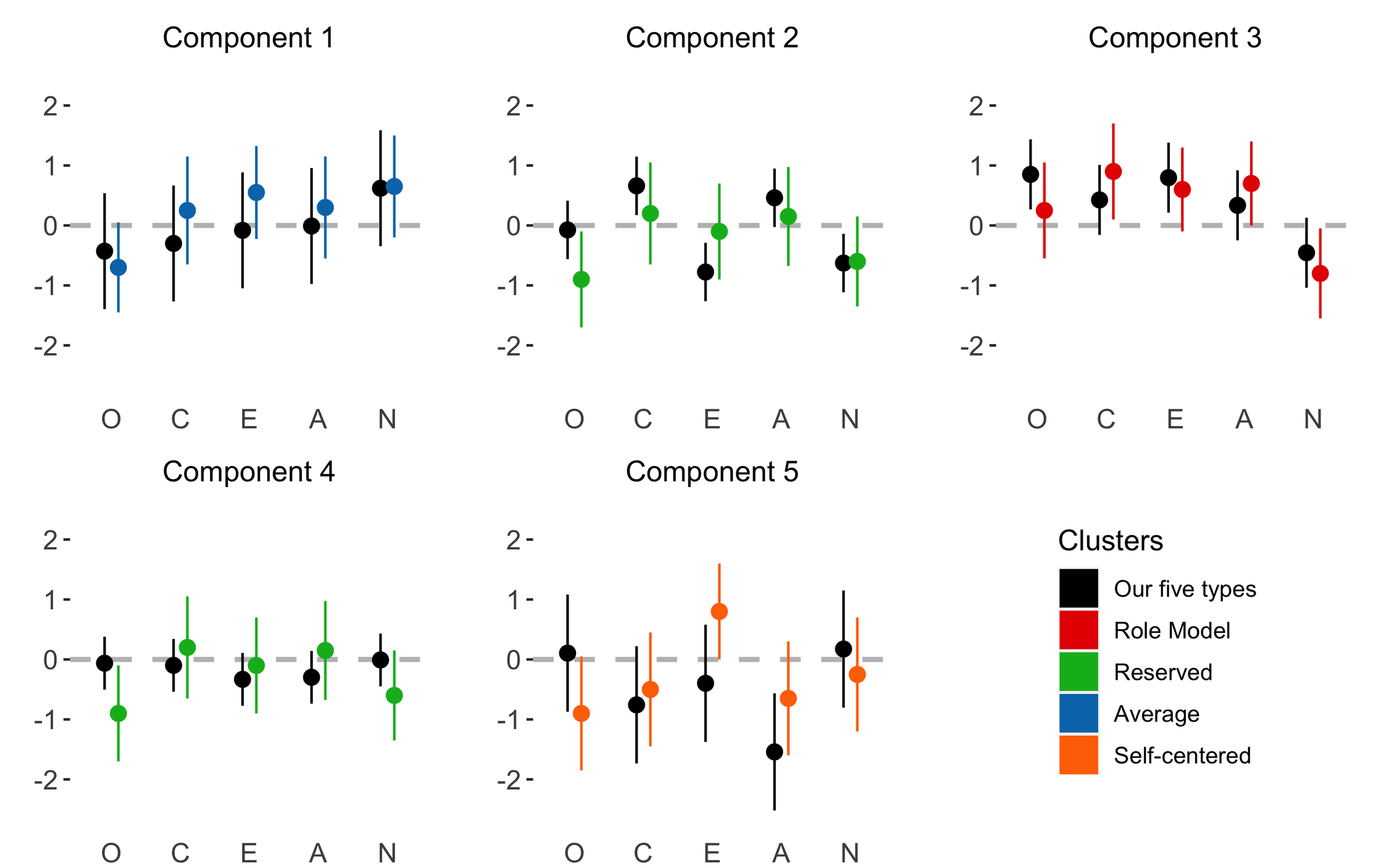

Supplement: S1 Fig — We select seven clusters thanks to the Likelihood Ratio Test (LRT = 16.39, p = 0.157) and choose to remove two clusters that represent less than 5% of bridge players (1.59% and 4.05%). Each of the five retained component is presented with its mean and standard deviation. Gerlach & al.’s types have been graphically estimated. Components are presented with the closest (minimum euclidean distance) Gerlach & al.’s type. The five personality traits are O: Openness, C: Conscientiousness, E: Extraversion, A: Agreeableness and N: Neuroticism. (TIF) [file pone.0305985.s007.tif]
